# Antiplatelet therapy adjustment improved the radiomic characteristics of acute silent cerebral infarction after stent-assisted coiling in patients with high on-treatment platelet reactivity: A prospective study

**DOI:** 10.3389/fnins.2023.1068047

**Published:** 2023-02-09

**Authors:** Wenqiang Li, Anxin Wang, Chao Ma, Yanmin Wang, Yapeng Zhao, Yisen Zhang, Kun Wang, Ying Zhang, Yang Wang, Xinjian Yang, Jian Liu, Xianzhi Liu

**Affiliations:** ^1^Department of Neurosurgery, The First Affiliated Hospital of Zhengzhou University, Zhengzhou, China; ^2^Department of Interventional Neuroradiology, Beijing Neurosurgical Institute and Beijing Tiantan Hospital, Capital Medical University, Beijing, China; ^3^China National Clinical Research Center for Neurological Diseases, Beijing Tiantan Hospital, Capital Medical University, Beijing, China; ^4^Department of Thoracic Surgery, The First Affiliated Hospital of Zhengzhou University, Zhengzhou, China; ^5^Department of Neurosurgery, Beijing Chaoyang Hospital, Capital Medical University, Beijing, China

**Keywords:** unruptured intracranial aneurysms, radiomics, stent placement, acute silent cerebral infarction, high on-treatment platelet reactivity, antiplatelet treatment adjustment

## Abstract

**Background:**

We aimed to investigate the effects of high on-treatment platelet reactivity (HPR) and antiplatelet therapy adjustment on high-risk radiomic features in patients with antiplatelet therapy adjustment on acute silent cerebral infarction (ASCI) who had unruptured intracranial aneurysms (UIA) after stent placement.

**Methods:**

This single-institution study prospectively included 230 UIA patients who had ACSI after stent placement in our hospital between January 2015 and July 2020. All patients underwent magnetic resonance imaging with diffusion-weighted imaging (MRI-DWI) after stent placement and 1,485 radiomic features were extracted from each patient. The least absolute shrinkage and selection operator regression methods were used for selection of high-risk radiomic features associated with clinical symptoms. In addition, 199 patients with ASCI were classified into three groups: controls without HPR (*n* = 113), HPR patients with standard antiplatelet therapy (*n* = 63) and HPR patients with antiplatelet therapy adjustment (*n* = 23). We compared high-risk radiomic features between three groups.

**Results:**

Of the patients who had acute infarction after MRI-DWI, 31 (13.5%) exhibited clinical symptoms. Eight risk radiomic features associated with clinical symptoms were selected, and the radiomics signature exhibited good performance. In ASCI patients, compared with controls, the radiomic characteristics of ischemic lesion in HPR patients were consistent with the following high-risk radiomic features associated with clinical symptoms: higher gray-level values, greater variance in intensity values, and greater homogeneity. However, the adjustment of antiplatelet therapy in HPR patients modified the high-risk radiomic features, which showed lower gray-level values, less variance in intensity values, and more heterogeneous texture. The radiomic shape feature of elongation showed no notable difference between three groups.

**Conclusion:**

Adjustment of antiplatelet therapy might reduce the high-risk radiomic features of UIA patients with HPR after stent placement.

## Introduction

Standard dual antiplatelet therapy (100 mg aspirin and 75 mg clopidogrel daily) plays a central role in the treatment of patients with unruptured intracranial aneurysms (UIAs) undergoing stenting treatment (Thompson et al., 2015; Walcott et al., 2016). However, a considerable proportion of patients develop high on-treatment platelet reactivity (HPR) during standard antiplatelet preparation, exhibiting an increased risk of thromboembolic events (Yang et al., 2016; Kim et al., 2018; Li et al., 2021). Several previous studies have reported that adjustment of antiplatelet therapy for patients with HPR can reduce the risk of thromboembolic complications, however, the thromboembolic complications of the patients were clinical symptoms (Hwang et al., 2015; Cagnazzo et al., 2019; González et al., 2020). Furthermore, clinical acute silent cerebral infarction (ASCI) is reported to exhibit higher incidence after endovascular embolization, ranging from 10 to 81.6% (Iosif et al., 2015; Park et al., 2016; Bond et al., 2017), and patients with ASCI have a higher rate of developing new neurological symptoms and cognitive impairment (Kang et al., 2013; Tan et al., 2015).

Magnetic resonance imaging with diffusion-weighted imaging (MRI-DWI) is reported to have high sensitivity and specificity for diagnosing acute ischemic stroke (González et al., 1999; Crisostomo et al., 2003; Vedel et al., 2018). The delayed thromboembolic events of patients with UIA after embolization in the early period are associated with platelet reactivity (Hwang et al., 2014; Kim et al., 2017). A previous study attempted to assess the relationship between HPR and thromboembolic complications in patients with intracranial aneurysm undergoing stent treatment, and found that DWI-positive ischemic cerebral lesions were more often detected in HPR patients (Yang et al., 2016). However, it is unclear whether HPR has any impact on the imaging characteristics of ASCI, or if the adjustment of antiplatelet therapy for HPR patients can improve imaging outcomes. Traditional visual inspection of DWI images and averaging intensity levels in large regions of interest often ignores subtle changes. However, radiomics analysis is a newly emerging field of study involving computer-based extraction of a large number of quantitative features, and can capture microscale information that is hidden in conventional imaging and not visible to the naked human eye (Bonekamp et al., 2018; Zinn et al., 2018).

In the current study, we used MRI-DWI images to extract radiomic features from the ischemic lesions associated with the presence of clinical symptoms. We then compared the extracted high-risk radiomic features of new ASCI between patients without HPR and patients with HPR undergoing stent placement for UIA, and evaluated the effects of adjusted antiplatelet preparation on the high-risk radiomic features in patients with HPR, compared with HPR patients without antiplatelet adjustment.

## Materials and methods

### Study participants

**FIGURE 1:**
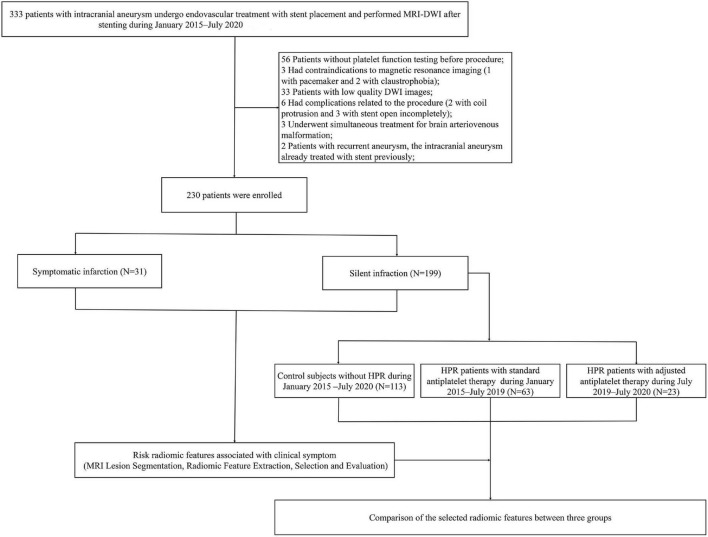
Flowchart of patient selection.

The study cohort was derived from two prospective study at our hospital (NCT03989557 and NCT02224131), patients diagnosed with UIA who were undergoing endovascular treatment with stent placement from January 2015 to July 2020 were enrolled. The study was approved by the Ethics Committee of our hospital, and written informed consent was obtained from all patients. The study was reported in line with the STROCSS criteria (Agha et al., 2019). Inclusion criteria were: (1) the patient had unruptured intracranial aneurysm and were undergoing stent placement; (2) new ischemic lesions were identified after stent placement (DWI was performed within 1 week after the procedure); and (3) patient’s age ≥ 18 years. Exclusion criteria were: (1) any contraindications for MRI (pacemakers, ferromagnetic implants or foreign bodies, and claustrophobia); (2) low-quality DWI images affected by image artifacts; (3) no platelet function testing before procedure; (4) complications related to the procedure or material quality; (5) undergoing simultaneous treatment for other cerebrovascular diseases; (6) a history of hypersensitivity to aspirin, clopidogrel, or ticagrelor; (7) prior treatment using an endovascular stenting approach for an intracranial aneurysm; (8) pregnant or lactating; and (9) malignant diseases, such as liver disease, kidney disease, congestive heart failure, and malignant tumors. The flowchart is shown in Figure 1. We identified 333 patients with UIA who underwent MRI-DWI after stent placement. After applying the exclusion criteria, a total of 230 patients (median age, 55.84 years; IQR, 18–77 years; 148 women) were enrolled in this study. Of the patients, 199 had ASCI, and another 31 had neurological symptomatic infarction. Time of symptomatic occurrence was before the performance of MRI-DWI in patients with clinical symptoms. At our center, we reached consensus about adjustment of antiplatelet therapy for patients with HPR in July 2019, at which time we began to implement this treatment strictly for patients with HPR. Thus, we classified patients with ASCI into three groups according to established diagnostic criteria of HPR and adjustment of antiplatelet therapy: control subjects without HPR (*n* = 113), HPR patients with standard antiplatelet therapy (*n* = 63) and HPR patients who underwent antiplatelet therapy adjustment (*n* = 23). Stenting procedures was showed in Supplementary Table 1. For light transmission aggregometry, 5 μmol/L adenosine diphosphate was used; 1 mg/ml arachidonic acid was used to evaluate the effects of acetylsalicylic acid specifically and sensitively on platelets. Maximal platelet aggregation (MPA) was defined as the percentage change in light transmittance. High on-treatment platelet reactivity was defined as follows: >50% MPA response to adenosine diphosphate and >20% MPA response to arachidonic acid (Griessenauer et al., 2019). For thromboelastography, HPR was defined as an arachidonic acid-induced platelet inhibition rate <50%, and an adenosine diphosphate -induced platelet inhibition rate <30% (Yang et al., 2016). For patients with HPR on clopidogrel after July 2019, clopidogrel 75 mg was switched to one dose of ticagrelor 180 mg before the procedure, followed by two daily doses of 90 mg of ticagrelor after the procedure. For patients with HPR on aspirin, the aspirin dose was increased to 200 mg. The treatment adjustment was administered at least 1 day before stenting.

### Image acquisition and MRI lesion segmentation

All patients underwent a conventional brain MRI protocol including DWI and axial, coronal, and sagittal T1- and T2-weighted images. The images were acquired using a 1.5 T scanner (Achieva, Philips Electronics Inc., Netherlands) in 66 patients, a 3.0 T/1.5 T scanner (Signa HDxt; GE Medical System, Milwaukee, WI, USA) in 97 patients, a 3.0 T scanner (Trio-Tim, Siemens, Erlangen, Germany), and a 3.0 T Siemens Skyra scanner (Siemens Medical Systems, Erlangen, Germany) in 67 patients. A detailed description of data preprocessing and MRI protocol is shown in Supplementary Table 2. Only DWI images were used in this study. DWI data were acquired using a single-shot twice-refocused spin-echo diffusion echo-planar imaging sequence. Sixty-four non-linear diffusion directions with *b* = 1,000 s/mm^2^ and an additional volume with *b* = 0 s/mm^2^, for both datasets, apparent diffusion coefficient maps were computed. The segmentation of the infarct lesion volume was performed by reader 1 (J L, with 10 years of experience in brain imaging) on the DWI computed with the *b* value = 1,000 s/mm2, where the infarct lesion was the most visible. The infarct lesion of DWI images were manually segmented for each patient using 3D Slicer (version 4.10.2) (Fedorov et al., 2012).

### Radiomic feature extraction, selection and evaluation

**FIGURE 2:**
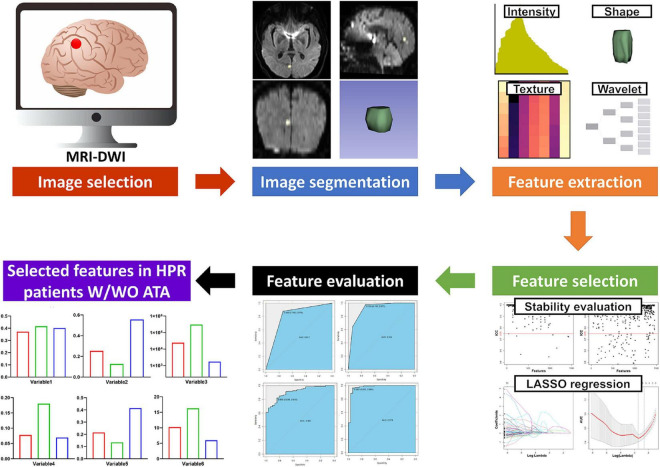
The radiomics workflow of the study.

The radiomics workflow is shown in Figure 2. A large number of radiomic features were automatically extracted from the segmented infarct lesion using pyradiomics (version 2.2.0) (Hofmeister et al., 2020). This allowed us to compute first-order statistics related to infarct lesion intensity, shape, and size features, including higher-order textural features using gray level co-occurrence matrix features, gray level size zone matrix features, gray level run length matrix, neighboring gray tone difference matrix, and gray level dependence matrix features. Further higher-order features were added by applying filters to the native MRI-DWI images (see Supplementary Table 3 for details on image filters used). Overall, a total of 1,485 radiomic features were extracted from each infarct lesion segmented on MRI-DWI.

To assess the stability of feature extraction, the reproducibility of intra-observer and inter-observer agreement for radiomics features was measured. Reader 1 repeated infarction segmentation twice in a 1-week period and reader 2 (XJ Y, with 20 years of experience in brain imaging) independently performed the segmentation. The intraclass correlation coefficient (ICC) was used to evaluate intra-observer and inter-observer agreement. An ICC score greater than 0.75 was considered to indicate satisfactory agreement. To guarantee the repeatability of the results, *z*-score normalization was performed as a data preprocessing step (Supplementary Table 4).

To avoid the curse of dimensionality and reduce the bias from radiomics features, three steps were adopted to select the features. First, all features were tested using an independent samples *t*-test or Mann–Whitney *U* test to select potential important features. Features that did not meet the criteria of either of the above tests were excluded. Second, the least absolute shrinkage and selection operator (LASSO) was used for dimensionality reduction and feature selection by performing variable selection and regularization to enhance the prediction accuracy and interpretability of the statistical radiomic features produced (Chen et al., 2019; Ji et al., 2020). The 1–standard error of the minimum criteria was used to tune the regularization parameter (λ) and for feature selection using 10-fold cross-validation, to construct a radiomics signature. Finally, the evaluation of the optimal radiomics signature mainly relied on the area under the receiver operating characteristic curve (AUC).

### Statistical analysis

All statistical analyses were conducted using R (Version 3.4.1) and GraphPad Prism (version 9.0). Clinical characteristics were measured based on the variable type. Continuous variables are presented as the means or medians and were compared using independent *t*-tests or Wilcoxon rank sum tests based on their distributions. Categorical variables were measured as proportions and were compared using chi-square tests or Fisher’s exact tests. *Post hoc* multiple comparisons were conducted using Tukey *post hoc* or Bonferroni comparisons. Pearson correlation was performed to assess the association of clinical symptoms and radiomic features. LASSO regression based on multivariate binary logistic regression was performed using the “glmnet” package. The correlation coefficient matrix was plotted using the “ggplot2” package. SVM, randomForest, adaboost models and ROC curves were created using the “e1071,” “randomForest,” “adabag,” and “pROC” packages, respectively. Differences were considered significant at *p* < 0.05.

## Results

### Patient characteristics between patients with ASCI and patients with symptomatic infarction, and between non-HPR patients, and HPR patients with/without antiplatelet adjustment

The baseline demographic and clinical characteristics of the patients are summarized in Supplementary Table 5. The proportion of HPR was significantly higher in patients with clinical symptoms compared with ASCI patients (83.9% vs. 43.2%, *p* < 0.001). There were no significant differences regarding other clinical and demographic variables between ASCI patients and patients with symptomatic infarction (all *p* > 0.05).

The baseline demographic and clinical characteristics of the 199 patients with ASCI are summarized in Table 1. Differences regarding clinical and demographic variables were assessed in non-HPR patients, HPR patients without antiplatelet adjustment and HPR patients with antiplatelet adjustment. None of the assessed variables showed significant differences between the three groups (all *p* > 0.05).

**TABLE 1:** Baseline characteristics of ACSI patients with non-HPR, HPR without antiplatelet adjustment, and HPR with antiplatelet adjustment.

Characteristic	Non-HPR (*N* = 113)	HPR without antiplatelet adjustment (*N* = 63)	HPR with antiplatelet adjustment (*N* = 23)
Age, years	56.35 ± 9.90	55.95 ± 10.24	53.61 ± 11.71
Female	72 (63.7)	40 (63.5)	15 (65.2)
Smoking	19 (16.8)	16 (25.4)	5 (21.7)
Drinking	18 (15.9)	12 (19.0)	3 (13.0)
Hypertension	49 (43.4)	25 (39.7)	11 (47.8)
Hyperlipidemia	45 (40.2)	33 (52.4)	12 (52.2)
DAPT, days	4.16 ± 1.41	3.97 ± 1.18	3.96 ± 1.66
Maximum size, mean (SD), mm	7.40 ± 5.91	8.01 ± 5.72	6.84 ± 5.10
Neck size, mean (SD), mm	5.21 ± 4.00	5.05 ± 3.72	4.93 ± 2.83
**Location**			
Anterior cerebral artery	16 (14.2)	6 (9.5)	4 (17.4)
Internal carotid artery	75 (66.4)	40 (63.5)	11 (47.8)
Middle cerebral artery	9 (8.0)	8 (12.7)	5 (21.7)
Posterior circulation	13 (11.5)	9 (14.3)	3 (13.0)
Flow diverter	15 (13.3)	12 (19.0)	6 (26.1)
Procedure time, h	1.92 ± 0.70	1.84 ± 0.61	2.09 ± 0.94
**Immediate occlusion grade**			
Complete	100 (88.5)	51 (81.0)	18 (78.3)
Residual neck	10 (8.8)	5 (7.9)	3 (13.0)
Residual sac	3 (2.7)	7 (11.1)	2 (8.7)
Time of DWI, days	1.25 ± 0.56	1.16 ± 0.45	1.13 ± 0.34

ASCI, acute silent cerebral infarction; DAPT, dual antiplatelet therapy; DWI, diffusion-weighted imaging; HPR, high on-treatment platelet reactivity.

### Selection of the best radiomic features between patients with ASCI and patients with symptomatic infarction

Satisfactory inter- and intra-observer reproducibility was achieved for radiomics feature extraction (Supplementary Figure 1). LASSO regression was used to select the eight best features to derive a radiomics signature (Supplementary Figure 2 and Supplementary Table 6), and the detailed process of radiomic feature selection is shown in Supplementary Table 7. The definitions and formulae of the eight selected radiomics signatures according to PyRadiomics and IBSI recommendations are presented in Supplementary Tables 8, 9. The comparison of the selected high-risk radiomic features between ASCI and patients with clinical symptoms is shown in Table 2. Six of the eight significant radiomic features selected were higher in patients with symptomatic infarction compared with patients with ASCI: Shape_Elongation, Firstorder_Total Energy (TE), Gray Level Size Zone Matrix_Small Area Emphasis (GLSZM_SAE), Firstorder_Range, Gray Level Co-occurrence Matrix_Difference Entropy (GLCM_DE), and Gray Level Dependence Matrix_Large Dependence High Gray Level Emphasis (GLDM_LDHGLE). Thus, ischemic lesions of patients with clinical symptoms were positively associated with greater elongation, higher gray-level values, greater variance in intensity values, and greater homogeneity of the ischemic texture (Supplementary Table 10). In contrast, two radiomic features were significantly higher in ASCI patients: GLSZM_Low Gray Level Zone Emphasis (GLSZM_LGLZE), and Gray Level Run Length Matrix_Gray Level Non-uniformity Normalized (GLRLM_GLNN). Patients with symptomatic infarction were negatively associated with lower gray-level values and more heterogeneity of the ischemic texture (Supplementary Table 10). Good performance of the radiomics signature was observed with multivariable logistic regression, support vector machine and random forest, and the AUC values were 0.907, 0.974 and 0.914, respectively (Supplementary Figure 3).

**TABLE 2:** Comparison of eight selected radiomics features after dimensionality reduction and feature selection using least absolute shrinkage and selection operator (LASSO) regression in patients with symptomatic infarction and ASCI.

Radiomics characteristics	Patients with symptomatic infraction (*n* = 31)	Patients with ASCI (*n* = 199)	*P*-value
Shape_Elongation	0.55 ± 0.22	0.39 ± 0.21	<0.001
GLSZM_LGLZE	0.13 ± 0.12	0.25 ± 0.17	<0.001
Firstorder_TE	1,191,371.33 ± 1,753,542.16	111,004.36 ± 363,121.83	0.002
GLSZM_SAE	0.17 ± 0.16	0.11 ± 0.12	0.013
GLRLM_GLNN	0.16 ± 0.82	0.21 ± 0.12	0.004
Firstorder_Range	16.39 ± 4.97	11.66 ± 4.14	<0.001
GLCM_DE	1.14 ± 0.24	1.01 ± 0.23	0.004
GLDM_LDHGLE	59.70 ± 14.14	41.14 ± 14.04	<0.001

ASCI, acute silent cerebral infarction; GLSZM, gray level size zone matrix; LGLZE, low gray level zone emphasis; TE, total energy; SAE, small area emphasis; GLRLM, gray level run length matrix; GLNN, gray level non-uniformity normalized; GLCM, gray level co-occurrence matrix; DE, difference entropy; GLDM, gray level dependence matrix; LDHGLE, large dependence high gray level emphasis.

### Comparison of the best radiomic features between non-HPR patients, HPR patients with/without antiplatelet adjustment

**FIGURE 3:**
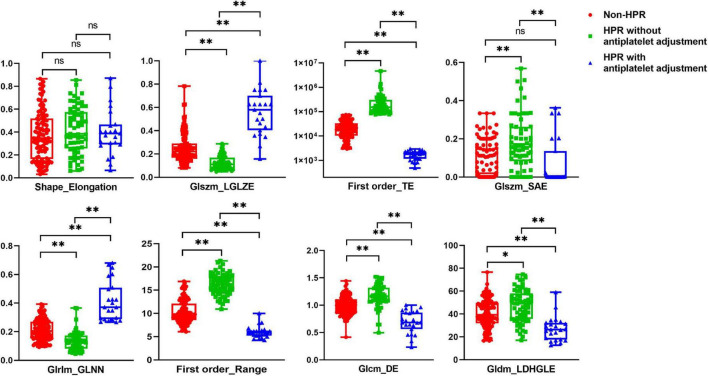
Comparison of the eight selected radiomics features in patients with non-high on-treatment platelet reactivity (HPR), HPR without antiplatelet adjustment, and HPR with antiplatelet adjustment. *p < 0.05, **p < 0.01.

The selected high-risk radiomic features were compared in the non-HPR patients, HPR patients without antiplatelet adjustment and HPR patients with antiplatelet adjustment (Figure 3 and Supplementary Table 1). Seven of the eight selected high-risk radiomic features exhibited significant differences between the three groups: GLSZM_LGLZE, Firstorder_TE, GLSZM_SAE, GLRLM_GLNN, Firstorder_Range, GLCM_DE, and GLDM_LDHGLE. Compared with the seven significant radiomic features in non-HPR patients, the HPR patients without antiplatelet adjustment had higher Firstorder_TE, Glszm_SAE, Firstorder_Range, Glcm_DE, and Gldm_LDHGLE, and lower Glszm_LGLZE and Glrlm_GLNN. Hence, the radiomic characteristics of ischemic lesion in HPR patients without antiplatelet adjustment were higher gray-level values, greater variance in the intensity values, and greater homogeneity compared with those of non-HPR patients, which is similar to the radiomic characteristics of patients with symptomatic infarction.

Furthermore, we compared the seven significant high-risk radiomic features between HPR patients with and without antiplatelet adjustment (Figure 3 and Supplementary Table 9). The values of GLSZM _LGLZE and GLRLM _GLNN were larger in HPR patients with antiplatelet adjustment compared with those in patients without antiplatelet adjustment, whereas the values of Firstorder_TE, GLSZM _SAE, Firstorder_Range, GLCM_DE, and GLDM_LDHGLE were lower. Thus, lower gray-level values, less variance in the intensity values, and a more heterogeneous texture were found in HPR patients with antiplatelet adjustment, which was the opposite pattern of results to the high-risk radiomic features of patients with symptomatic infarction. However, the radiomic shape feature of elongation showed no notable difference between the three groups.

## Discussion

### Principal findings

Among UIA patients with new ischemic lesions after stent placement, patients with HPR to antiplatelet therapy had a greater risk of symptomatic infarction. We proposed that the selected eight best radiomic features between patients with ASCI and patients with clinical symptoms were high-risk radiomic features. High-risk radiomic factors in patients with symptomatic infarction were associated with greater elongation, higher gray-level values, greater variance in intensity values, and more homogenous ischemic texture. In patients with ASCI after stent placement, although these patients did not suffer from clinical neurological symptoms, the radiomic features of HPR patients without antiplatelet adjustment showed higher gray-level values, greater variance in intensity values, and more homogeneity than non-HPR patients, who exhibited higher levels of high-risk radiomic features associated with clinical neurological symptoms. However, with antiplatelet therapy adjustment in HPR patients, the high-risk radiomic features associated with neurological symptoms were modified, exhibiting lower gray-level values, less variance in the intensity values, and a more heterogeneous texture compared with HPR patients without antiplatelet therapy adjustment. The results suggest beneficial effects of antiplatelet therapy adjustment in HPR patients with ASCI, with a decrease in the risk of radiomic characteristics associated with clinical neurological symptoms.

### Clinically silent infarction associated with cognitive decline in UIA patients after embolization

In UIA patients with embolization, unexpected ASCI is frequently reported (Kang et al., 2013; Iosif et al., 2015; Park et al., 2016; Yang et al., 2016). Bond et al. (2017) performed a systematic review and meta-analysis to study the incidence of positive DWI findings for thromboembolic events following endovascular treatment of intracranial aneurysms. They found that the overall incidence of positive DWI findings for thromboembolic events was 49% following coil embolization, and 43% following stent-assisted coiling. The incidence of ASCI was remarkably high in UIA patients with endovascular treatment, ranging from 10 to 81.6%, and several studies reported that ASCI can lead to long term cognitive decline and neurodegeneration (Vermeer et al., 2003; Debette et al., 2010; Fanning et al., 2014). However, several other studies suggested that endovascular coiling for UIA patients does not diminish neurocognitive function, and cognitive function of the patients with ASCI exhibited recovery or improvement from baseline cognitive function after 4 weeks (Kang et al., 2013; Srivatsan et al., 2021). However, an important limitation of these studies is that the sample sizes were relatively small, decreasing the statistical power of the studies and making it hard to generalize their conclusions to other patients. A recent prospective randomized-controlled trial found that the patients with iatrogenic infarcts have worse post-procedural outcomes (Ganesh et al., 2022). Similarly, substantial evidence regarding cardiac disease and treatment procedures has indicated that ASCI is often described as a precursor to symptomatic stroke and is associated with cognitive decline, dementia, and depression (Auffret et al., 2016; Chen et al., 2019; Conen et al., 2019).

### Effect of HPR and antiplatelet adjustment in thromboembolic complications of UIA patients after embolization

There are conflicting results in the literature regarding the role of platelet activity. Several clinicians and scholars have endorsed evaluating platelet activity in patients treated with antiplatelet therapy, proposing that adjusting antiplatelet therapy for HPR patients might reduce the number of thromboembolic events (Yang et al., 2016; Adeeb et al., 2017). Other studies, however, reported conflicting results, reporting that platelet function testing and personalized antiplatelet therapy in patients undergoing stenting did not reduce thrombotic complications (Brinjikji et al., 2015; Neyens et al., 2020). Retrospective designs represent a major limitation of many previous studies, and few prospective randomized studies have been conducted. Previously, we conducted a randomized controlled trial to explore whether monitoring platelet function for the purpose of adjusting antiplatelet therapy would improve clinical outcomes in UIA patients with HPR after stent placement (Li et al., 2021). The results revealed that patients with HPR to aspirin and clopidogrel might experience a greater risk of thromboembolic events, and adjustment of antiplatelet therapy based on the platelet function test might aid in the reduction of thromboembolic events, which was consistent with the results of other prospective studies (Hwang et al., 2015; Kim et al., 2017). However, in these studies, thromboembolic events were defined as infarction with clinical symptoms. Hwang et al. (2015) and Li et al. (2021) proposed that silent infarction in UIA patients after embolization might be worth investigating in future studies, because silent infarction might cause cognitive impairment and a higher rate of developing new neurological symptoms.

The association between silent embolic cerebral infarction and platelet reactivity after endovascular treatment was explored in several previous studies (Kim et al., 2014; Iosif et al., 2015; Yang et al., 2015; Park et al., 2016; Tokunaga et al., 2019). These studies evaluated the relationship between platelet function and acute embolic complications in UIA patients undergoing endovascular treatment, and reported that platelet function was not associated with postprocedural ASCI in endovascular treatment for unruptured cerebral aneurysms (Iosif et al., 2015; Park et al., 2016; Tokunaga et al., 2019). Conversely, Yang et al. (2015) concluded that the antiplatelet inhibition parameter was a predictor for ASCI in unruptured intracranial aneurysm patients treated with stent-assisted coiling. One plausible hypothesis to explain these conflicting findings is that platelet reactivity after dual antiplatelet treatment measured before stenting may not predict ASCI, and increased platelet activation after endovascular procedure might be important in ASCI. However, aspirin and clopidogrel were administered only 1 day before stenting, and loading doses of antiplatelet agents were administered in one study (Kim et al., 2014). The blood drug concentration of aspirin and clopidogrel might not reach a stable concentration or stable effects within such a short period of time. At our center, we typically perform antiplatelet therapy at least 5 days before a stenting procedure, and this factor may have led to discrepancies in previous findings. Furthermore, previous studies of platelet function and ASCI have typically focused only on qualitative factors, without quantitative analysis of imaging data from the ASCI lesion. Radiomics is a data-centric approach for extracting quantitative and reproducible information to quantify the tissue and lesion properties radiographic phenotypes (Mayerhoefer et al., 2020). With the aid of radiomics tools, more intrinsic information can be extracted from imaging data sets. This tool enables assessment of the effects of HPR and antiplatelet therapy adjustment for UIA patients with ASCI detected with MRI-DWI.

### Radiomic characteristics of MRI-DWI in UIA patients with ASCI undergoing stent placement

Kahlert et al. (2012) proposed that it is not the number of new lesions but the size of the ASCI lesion that is related to the incidence of clinical stroke. Furthermore, Wiśniewski et al. (2020) found a positive relationship between platelet reactivity and acute ischemic focus volume, in which individuals with HPR were significantly more likely to have a large ischemic focus among patients with large-vessel disease. However, besides the infarction lesion size, the relationships between HPR and other imaging characteristics, including texture, shape, and higher-order parameters, have not been examined in previous studies. In the current study, we found that the infarction lesions of patients with clinical symptoms were positively associated with higher elongation, higher gray-level values, more variance in intensity values, and more homogeneity, and were negatively associated with lower gray-level values and more heterogeneity. Compared with the high-risk radiomic characteristics associated with clinical symptoms between patients with and without HPR, the radiomic characteristics of HPR patients indicated a higher risk than those without HPR, exhibiting higher gray-level values, greater variance in the intensity values, and greater homogeneity. Furthermore, we found that adjustment of antiplatelet therapy for HPR patients reduced the high-risk radiomic characteristics associated with clinical symptoms. Therefore, although patients with ASCI did not develop clinical neurological symptoms, HPR to antiplatelet therapy had an influence on the characteristics of MRI-DWI imaging, which presented with high-risk radiomic features associated with symptomatic infarction. However, the high-risk radiomic features of ASCI lesions were modified by adjustment of antiplatelet therapy in HPR patients, with a reduction in the risk of radiomic characteristics associated with neurological symptoms. These findings suggest that individuals with HPR had more high-risk radiomic factors associated with symptomatic infarction in UIA patients with ASCI after stent placement, and that patients with HPR could benefit from the effects of the adjustment of antiplatelet therapy.

### Limitations

This study had several limitations. First, this was a single-center, observative study. A single-center design was selected because of the need to control for important confounding factors (e.g., patient selection and stenting techniques). Second, the sample size of HPR patients with antiplatelet therapy adjustment was small relative to the entire cohort, because the period of antiplatelet adjustment was relatively short in this study. Third, because patients were recruited at different time periods, there was variability in the period and duration of endovascular treatment and MRI-imaging acquisition, which could produce certain bias. Fourth, the volumes of interest for infarction lesions were drawn manually. Thus, the selection of radiomic features is a time-consuming task, and may suffer from poor reproducibility. The IBSI recommendations might help ameliorate this limitation of poor reproducibility. The development of automated segmentation techniques would be useful in the future. Fifth, because histopathological assessment of infarction lesion was not systematically conducted, we were unable to directly link the radiomic features to the biopsy of infarction lesions. Sixth, computing *z*-scores for the test cohort using statistics from the development cohort may have resulted in a slightly optimistic assessment of model performance.

## Conclusion

An MRI-DWI-based radiomic model showed excellent performance in discriminating between ASCI and non-ASCI patients. In ASCI patients, patients with HPR exhibited an increased risk of radiomic features associated with clinical symptoms in patients with UIA after stent placement. Adjustment of antiplatelet therapy in patients with HPR was associated with a reduction of high-risk radiomic features. These results suggest that adjustment of antiplatelet therapy may be an attractive treatment option to reduce high-risk radiomic features for UIA patients with HPR after stent placement.

## Data availability statement

The raw data supporting the conclusions of this article will be made available by the authors, without undue reservation.

## Ethics statement

The studies involving human participants were reviewed and approved by the Ethics Committee of Beijing Tiantan Hospital. The patients/participants provided their written informed consent to participate in this study.

## Author contributions

WL and XL performed the manuscript writing. WL, CM, YW, YaZ, YisZ, KW, and YinZ acquired the data. WL, AW, YW, XL, JL, and XY contributed to data analysis and interpretation. JL, XY, YW, and XL contributed to the experimental design and manuscript revision, and handled funding and supervision. All authors contributed to the article and approved the submitted version.
