# Supplementary material for: Antiplatelet therapy adjustment improved the radiomic characteristics of acute silent cerebral infarction after stent-assisted coiling in patients with high on-treatment platelet reactivity: A prospective study
Source: Front Neurosci. 2023 Feb 9;17:1068047. doi: 10.3389/fnins.2023.1068047 (PMC9948085; doi:10.3389/fnins.2023.1068047)

**Table E1 Description of Stenting Procedures, Platelet Function Testing and Antiplatelet Treatment Adjustment**

| |
|---|
| All patients were treated with standard dual-antiplatelet therapy (100 mg aspirin and 75 mg clopidogrel) at least 5 days before the procedure. Platelet function tests (light transmission aggregometry and thromboelastography were conducted to identify the HPR, and the antiplatelet regimen was further adjusted in these patients. All procedures were performed under general anesthesia, and fully procedural heparinization was used to achieve a targeted activated clotting time of 250–300 s. Rotational angiography, followed by three-dimensional image reconstruction through volume rendering was performed before embolization in all patients. Based on the images generated using rotational acquisition, at least two working projections that provide the best achievable view of the aneurysm neck were defined. Stent-assisted coiling was employed for aneurysms that were unfavorable for standard coiling. Properly-shaped microcatheters were introduced over microguidewires and navigated into the aneurysm cavity under the guidance of the microguidewire. During the coiling procedure, a coil suitable for safe packing was selected among these coils at every step. Aneurysms were packed as densely as possible with coils. The jailing technique was the usual method, in which a stent was deployed after the microcatheter was in position, and the first coil was deployed but not detached. Furthermore, for complex UIA patients, a flow diverter was chosen for complete occlusion of UIAs. For patients receiving a flow diverter, we adopted a triaxial support system to access the aneurysm. The flow diverter was introduced through a microcatheter, delivered to the parent artery defect, then deployed. Several endovascular techniques (the use of wires, catheters, or balloon angioplasty) were performed if the device was inadequately expanded. For aneurysms treated with flow diverter and coiling, similar techniques were performed, such as stent-assisted coiling. A final postembolization angiography is performed in the working projection to evaluate the occlusion grade and detect any complications. Frontal and lateral projections are also acquired at the end of the procedure. |

**Table E2 Description of MRI protocol**

| **Data preprocessing** |
| --- |
| For data preprocessing, the DWI data was visually inspected for apparent artifacts arising from participant motion or instrument malfunction. Before segmentation of the infarct lesions, we performed conventional correction using the 'eddy correct' tool in FSL, which registers the DWI volumes to the first volume acquired with b = 0 s/mm2. Distortions in the DWI images caused by eddy currents and simple head motions were corrected by applying affine alignment. Considering that different imaging from different MRI machines, different scanners or different acquisition protocols, the range of pixel values of medical images might vary greatly and volumes of interest are common in heterogeneous voxel spacing. To reduce the side-effect of pixel value outliers, all the pixel values in each image were sorted and the intensities were truncate to the range of 0.5 to 99.5 percentiles. In addition, we employed spatial normalization to reduce the effect of voxel spacing variation. Fixed resolution resampling method was used to handle the aforementioned problems. |
| **MRI protocol** |
| Using a 1.5-T Philips scanner (Achieva, Philips Electronics Inc., Netherlands), the scanning sequences included sagittal T2-weighted (T2W) or T1-weighted (T1W) imaging, axial T1W and T2W imaging, with axial fluid attenuated inversion recovery (FLAIR) and diffusion-weighted imaging (DWI) sequences. Parameters were as follows: spin echo (SE) T1W imaging: time of repetition (TR) = 380–500 ms, echo time (TE) = 8–16 ms; fast spin echo (FSE) T2W imaging: TR = 4,200–4,500 ms, TE = 87.6–98.6 ms; FLAIR: TR = 9,002 ms, TE = 133 ms, TI= 2200 ms; DWI adopted spin echo, echo planar imaging (SE EPI) sequence scanning: b values = 0 and 1,000 s/mm$^2$, TR = 3,000–6,600 ms, TE = 74–100 ms. The field of view was within the range of 200–240 mm. Slice thickness was 1.0 mm, the interslice gap was 1.5 mm, and the matrix size was 512 × 512 or 256 × 256.<br><br>Using a 3.0 T GE scanner (Signa HDxt; GE Medical System, Milwaukee, WI), the scanning sequences included T1WI, T2WI, FLAIR, and DWI. The scanning parameters were FLAIR, TR/TE = 9,000 ms/140 ms, TI = 2,600 ms, and the turning angle was 15. Layer thickness: 6.5 mm; layer spacing: 1.3 mm; DWI (b = 0, l,000 s/mm$^2$), single excitation SE/EPI sequence, TR/TE = 2,400 ms/104 ms, field of view (FOV) 200 mm × 220 mm, matrix 128 × 128, slice thickness 6.5 mm, slice spacing 1.3 mm, layers from 17 to 18.<br><br>Using a 3T Siemens Skyra scanner (Siemens Medical Systems, Erlangen, Germany), the scanning sequences included T1WI, T2WI, FLAIR, DWI. The scanning parameters were as follows: TR/TE = 1,000/17, slice thickness = 0.5 mm, acquisition plane = sagittal, FOV = 18 × 18 cm, matrix = 360 × 360, echo train length = 60, echo spacing = 5.55 ms, echo train duration = 333 ms, bandwidth = 400 Hz/pixel, partial Fourier = 7/8, and GRAPPA factor = 2. DWI images were obtained using single-shot echo-planar imaging with 2 b-factors (0 and 1,000 s/mm$^2$). Apparent diffusion coefficient (ADC) maps were generated automatically and included both b values in a monoexponential decay model. |

**Table E3 Radiomic features and filters**

| Radiomics feature class | Number of radiomic features |
|---|---|
| First-order statistics | 18 |
| Shape based | 13 |
| Gray level co-occurrence matrix | 23 |
| Gray level run length matrix | 16 |
| Gray level size zone matrix | 16 |
| Neighboring gray tone difference matrix | 5 |
| Gray level dependence matrix | 14 |
| Image filters | |
| Laplacian of Gaussian (sigma: 0.5, 1.0, 2.0) | 276 |
| Wavelet | 736 |
| Square | 92 |
| Square root | 92 |
| Logarithm | 92 |
| Exponential | 92 |
| Total | 1,485 |

**Table E4. Protocol of Z-score normalization**

| Z-score normalization |
| --- |
| Different radiomics features have different value ranges, which makes it difficult to compare two features with variable orders of magnitude. Prior to further analysis, z-score normalization was employed to eliminate different feature magnitudes by scaling values to a mean of 0 and a standard deviation of 1 using the following formula: $$z = \frac{x-\mu}{\sigma}$$ where $\mu$ is the mean for the population and $\sigma$ is the standard deviation for the population. |

**Table E5.** Baseline characteristics of patients with a new ischemic lesion on MRI-DWI.

| Characteristic | All patients(n=230) | Patients with symptomatic infraction (n=31) | Patients with ASCI (n=199) | P value |
|---|---|---|---|---|
| Age, y | 55.84 ± 10.30 | 55.42 ± 11.05 | 55.91 ± 10.21 | 0.806 |
| Female | 148 (64.3) | 21 (67.7) | 127 (63.8) | 0.671 |
| Smoking | 47 (20.4) | 7 (22.6) | 40 (20.1) | 0.750 |
| Drinking | 35 (15.2) | 2 (6.5) | 33 (16.6) | 0.144 |
| Hypertension | 103 (48.8) | 18 (58.1) | 85 (42.7) | 0.266 |
| Hyperlipidemia | 102 (44.3) | 12 (38.7) | 90 (45.5) | 0.482 |
| DAPT, d | 4.10 ± 1.36 | 4.23 ± 1.33 | 4.08 ± 1.37 | 0.568 |
| HPR | 112 (48.7) | 26 (83.9) | 86 (43.2) | <0.001 |
| Maximum size, mean (SD), mm | 7.57 ± 5.65 | 7.86 ± 5.05 | 7.53 ± 5.74 | 0.760 |
| Neck size, mean (SD), mm | 5.10 ± 3.66 | 4.94 ± 2.76 | 5.12 ± 3.78 | 0.789 |
| Location | | | | 0.769 |
| Anterior cerebral artery | 29 (12.6) | 3 (9.7) | 26 (13.1) | |
| Internal carotid artery | 147 (63.9) | 21 (67.7) | 126 (63.3) | |
| Middle cerebral artery | 24 (10.4) | 2 (6.5) | 22 (11.1) | |
| Posterior circulation | 30 (13.0) | 5 (16.1) | 25 (12.6) | |
| Flow diverter | 42 (18.3) | 9 (29.0) | 33 (16.6) | 0.095 |
| Procedure time, h | 1.95 ± 0.91 | 2.22 ± 1.74 | 1.91 ± 0.70 | 0.084 |
| Immediate occlusion grade | | | | 0.093 |
| Complete | 191 (83.0) | 22 (71.0) | 169 (85.0) | |
| Residual neck | 22 (9.6) | 4 (12.9) | 18 (9.0) | |
| Residual sac | 17 (7.4) | 5 (16.1) | 12 (6.0) | |
| Time of DWI, d | 1.18 ± 0.48 | 1.06 ± 0.25 | 1.21 ± 0.50 | 0.128 |

ASCI, acute silent cerebral infarction; DAPT, dual antiplatelet therapy; HPR, high on-treatment platelet reactivity; DWI, diffusion-weighted imaging.

**Table E6. Information of eight selected radiomics features after dimensionality reduction and feature selection using LASSO regression.**

| **No** | Radiomic features |
|---|---|
| N1 | Original_Shape_Elongation |
| N94 | Original_Glszm_LGLZE |
| N123 | Log-sigma-0-5-mm-3D_Firstorder_TE |
| N1027 | Wavelet-HHH_Glszm_SAE |
| N1095 | Wavelet-LLL_Glrlm_GLNN |
| N1234 | Square root_Firstorder_Range |
| N1245 | Square root_Glcm_DE |
| N1268 | Square root_Gldm_LDHGLE |

**Table E7. The process of optimal radiomics feature selection**

| Radiomics feature selection process |
| --- |
| For features with unsatisfactory agreement (Supplementary Figure E1), the rate of satisfactory agreement for all 1,485 features reached 98.3% in intra-observer agreement, and 16 features were excluded. The rate of satisfactory agreement for all features reached 91.6% in inter-observer agreement, and 125 features were excluded. Finally, 141 features were excluded, and the remaining 1,344 features were included in further analyses. Satisfactory inter- and intra-observer reproducibility was achieved for radiomics feature extraction. Twenty-five features showed a Gaussian distribution with homoscedasticity, but none were found to be significant in independent samples t-tests. Mann–Whitney U tests showed that 1,319 features were significantly different ($p < 0.05$). Therefore, a total of 1,319 features were used for LASSO regression. Eight features were selected by LASSO, with the best tuned regularization parameter $\lambda$ of 0.001 under the minimum criteria found by 10-fold cross validation (Supplementary Figure E2, and Table E5). |

**Table E8. Definitions of significant radiomic features**

| Radiomics | Definition and formula |
|---|---|
| Elongation | Elongation shows the relationship between the two largest principal components in the ROI shape. For computational reasons, this feature is defined as the inverse of true elongation.<br>$$elongation = \sqrt{\frac{\lambda_{minor}}{\lambda_{major}}}$$<br>Here, λmajor and λminor are the lengths of the largest and second largest principal component axes. The values range between 1 (where the cross section through the first and second largest principal moments is circle-like (non-elongated)) and 0 (where the object is a maximally elongated: i.e., a one-dimensional line).<br>The principal component analysis was performed using the physical coordinates of the voxel centers defining the ROI. Thus, the analysis takes spacing into account but does not make use of the shape mesh. |
| Low gray level zone emphasis (LGLZE) | $$LGLZE = \frac{\sum_{i=1}^{N_g}\sum_{j=1}^{N_s}\frac{\mathbf{P}(i,j)}{i^2}}{N_z}$$<br>LGLZE measures the distribution of lower gray-level size zones, with a higher value indicating a greater proportion of lower gray-level values and size zones in the image. |
| Total energy (TE) | $$total\ energy = V_{voxel}\sum_{i=1}^{N_p}(\mathbf{X}(i)+c)^2$$<br>Here, c is an optional value, defined by voxelArrayShift, which shifts the intensities to prevent negative values in **X**. This ensures that voxels with the lowest gray values contribute the least to energy, instead of voxels with gray level intensity closest to 0.<br>Total energy is the value of the energy feature scaled by the volume of the voxel in cubic mm. |
| Small area emphasis (SAE) | $$SAE = \frac{\sum_{i=1}^{N_g}\sum_{j=1}^{N_s}\frac{\mathbf{P}(i,j)}{j^2}}{N_z}$$ |

| | |
|---|---|
| | SAE is a measure of the distribution of small size zones, in which a greater value is indicative of more smaller size zones and more fine textures. |
| Gray level non-uniformity normalized (GLNN) | $$GLNN = \frac{\sum_{i=1}^{N_g} \left(\sum_{j=1}^{N_s} \mathbf{P}(i,j)\right)^2}{N_z^2}$$ GLNN measures the similarity of gray-level intensity values in the image, where a lower GLNN value correlates with a greater similarity in intensity values. This is the normalized version of the GLN formula. |
| Range | $$range = \max(\mathbf{X}) - \min(\mathbf{X})$$ The range of gray values in the ROI. |
| Difference entropy (DE) | $$difference\ entropy = \sum_{k=0}^{N_g-1} p_{x-y}(k) \log_2 \left(p_{x-y}(k) + \epsilon\right)$$ Difference entropy is a measure of the randomness/variability in neighborhood intensity value differences. |
| Large dependence high gray level emphasis (LDHGLE) | $$LDHGLE = \frac{\sum_{i=1}^{N_g} \sum_{j=1}^{N_d} \mathbf{P}(i,j) i^2 j^2}{N_z}$$ LDHGLE measures the joint distribution of large dependence with higher gray-level values. |

**Table E9. Definitions of significant radiomic features according to the IBSI Recommendations.**

| Radiomics | Definition and formula |
|---|---|
| Elongation | The ratio of the major and minor principal axis lengths could be viewed as the extent to which a volume is longer than it is wide, i.e. is eccentric. For computational reasons, we express elongation as an inverse ratio. 1 is thus completely non-elongated, e.g. a sphere, and smaller values express greater elongation of the ROI volume. $$F_{morph.pca.elongation} = \sqrt{\frac{\lambda_{minor}}{\lambda_{major}}}$$ |
| Low gray level zone emphasis (LGLZE) | This feature is a grey level analogue to small zone emphasis. Instead of small zone sizes, low grey levels are emphasised. The feature is defined as: $$F_{szm.lgze} = \frac{1}{N_s}\sum_{i=1}^{N_g}\frac{s_{i.}}{i^2}$$ |
| Difference entropy (DE) | The difference entropy for the diagonal probabilities36 is defined as: $$F_{cm.diff.entr} = -\sum_{k=0}^{N_g-1} p_{i-j,k}\log_2 p_{i-j,k}$$ |
| Large dependence high gray level emphasis (LDHGLE) | **High dependence high grey level emphasis** The high dependence high grey level emphasis feature emphasises neighbouring grey level dependence counts in the lower right quadrant of the NGLDM, where high dependence counts and high grey levels are located. The feature is defined as: $$F_{ngl.hdhge} = \frac{1}{N_s}\sum_{i=1}^{N_g}\sum_{j=1}^{N_n} i^2 j^2 s_{ij}$$ |

**Table E10 The correlation of selected radiomic features and the ischemic lesions of patients with clinical symptoms**

| | Shape_ Elongation | Glszm_ LGLZE | Firstorder_ TE | Glszm_ SAE | Glrlm_ GLNN | Firstorder_ Range | Glcm_ DE | Gldm_ LDHGLE |
|---|---|---|---|---|---|---|---|---|
| PCC | 0.26 | -0.23 | 0.46 | 0.16 | -0.16 | 0.36 | 0.19 | 0.41 |
| Significance | <0.001 | <0.001 | <0.001 | 0.013 | 0.019 | <0.001 | 0.004 | <0.001 |

PCC, Pearson correlation coefficient.

**Table E11.** Post hoc multiple comparisons of the eight selected radiomics in non-HPR, HPR without antiplatelet adjustment and HPR with antiplatelet adjustment patients.

| **Characteristic** | Patients with symptomatic infraction (n=31) (A) | Non-HPR (N=113) (B) | P value (A vs B) | HPR without antiplatelet adjustment (N=63) (C) | P value (A vs C) | P value (B vs C) | HPR with antiplatelet adjustment (N=23) (D) | P value (A vs D) | P value (B vs D) | P value (C vs D) |
|---|---|---|---|---|---|---|---|---|---|---|
| Shape_Elongation | 0.55 ± 0.22 | 0.37 ± 0.22 | <0.001 | 0.42 ± 0.21 | 0.003 | 0.187 | 0.40 ± 0.20 | 0.010 | 0.547 | 0.774 |
| Glszm_LGLZE | 0.13 ± 0.12 | 0.25 ± 0.12 | <0.001 | 0.13 ± 0.06 | 1.000 | <0.001 | 0.56 ± 0.20 | <0.001 | <0.001 | <0.001 |
| Firstorder_TE | 1191371.33 ± 1753542.16 | 24344.33 ± 17962.69 | 0.005 | 306340.50 ± 603009.40 | 0.049 | 0.003 | 1717.72 ± 702.76 | 0.004 | <0.001 | 0.001 |
| Glszm_SAE | 0.17 ± 0.16 | 0.08 ± 0.10 | <0.001 | 0.18 ± 0.14 | 0.524 | <0.001 | 0.07 ± 0.13 | <0.001 | 0.761 | <0.001 |
| Glrlm_GLNN | 0.16 ± 0.82 | 0.22 ± 0.08 | 0.012 | 0.13 ± 0.07 | 0.519 | <0.001 | 0.42 ± 0.14 | <0.001 | <0.001 | <0.001 |
| Firstorder_Range | 16.39 ± 4.97 | 10.25 ± 2.51 | <0.001 | 16.27 ± 2.34 | 1.000 | <0.001 | 5.97 ± 1.30 | <0.001 | <0.001 | <0.001 |
| Glcm_DE | 1.14 ± 0.24 | 0.99 ± 0.17 | <0.001 | 1.17 ± 0.21 | 0.524 | <0.001 | 0.70 ± 0.21 | <0.001 | <0.001 | <0.001 |
| Gldm_LDHGLE | 59.70 ± 14.14 | 40.67 ± 27.13 | <0.001 | 47.11 ± 14.33 | <0.001 | 0.002 | 27.13 ± 10.61 | <0.001 | <0.001 | <0.001 |

**Figure E1.** Evaluation of feature stability and inter-observer and intra-observer agreement on the basis of interclass correlation coefficient (ICC). A. All features exhibited good intra-observer agreement with ICCs of > 0.75 (above the red cutoff line). B. All features exhibited good inter-observer agreement, with ICCs of > 0.75(above the red cutoff line).

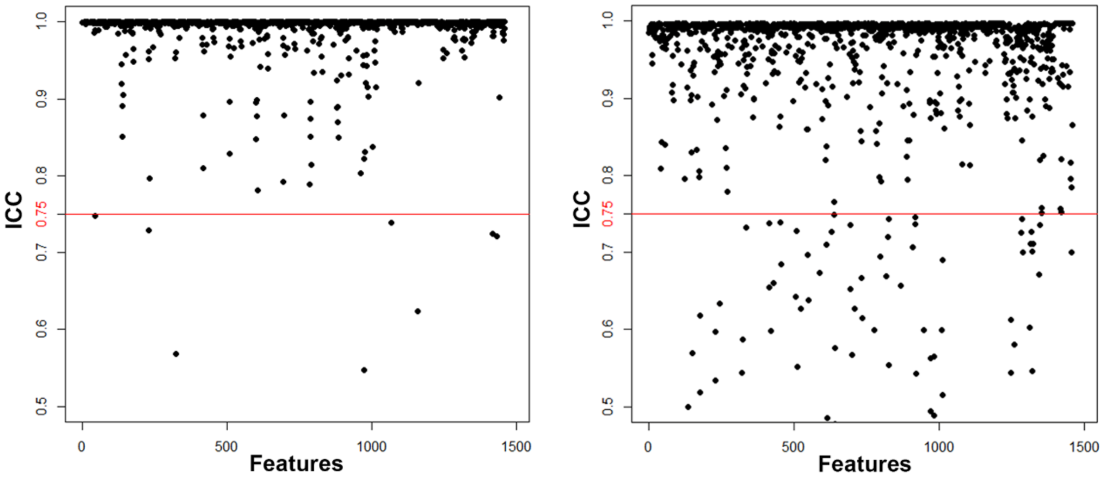

**Figure E2.** Feature selection using the least absolute shrinkage and selection operator (LASSO) regression. A, LASSO coefficient profiles for the 1,319 radiomics features. The vertical line shows the optimal value of λ (= 0.00984) and eight corresponding features with non-zero coefficients. B, The AUC curve was plotted by tuning parameter (λ) selection performed using 10-fold cross-validation. Dotted lines on the left and right denote the minimum criterion.

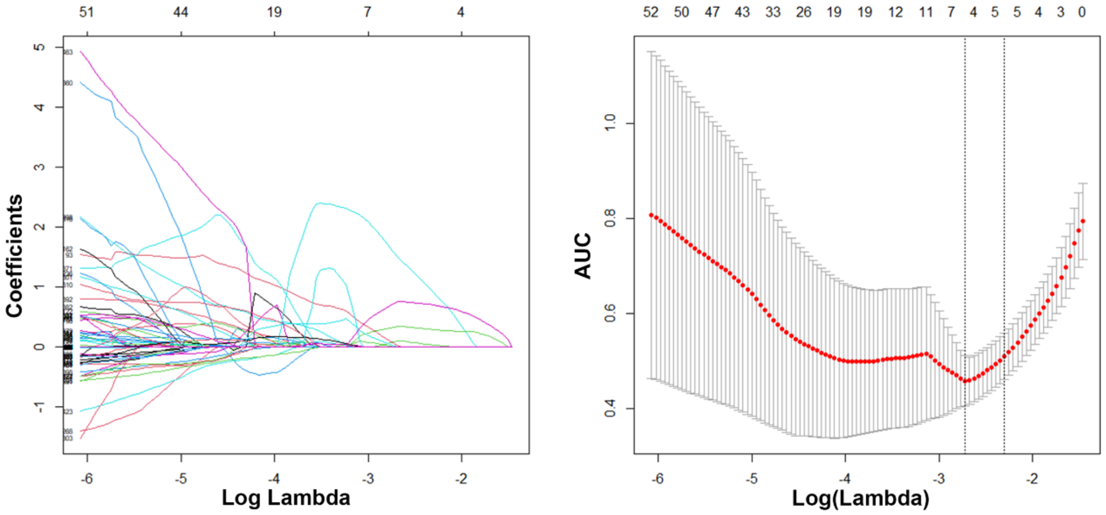

**Figure E3.** The receiver operating characteristic curve of multivariable logistic regression, support vector machine and random forest models. The area under the curve (AUC) value was provided.

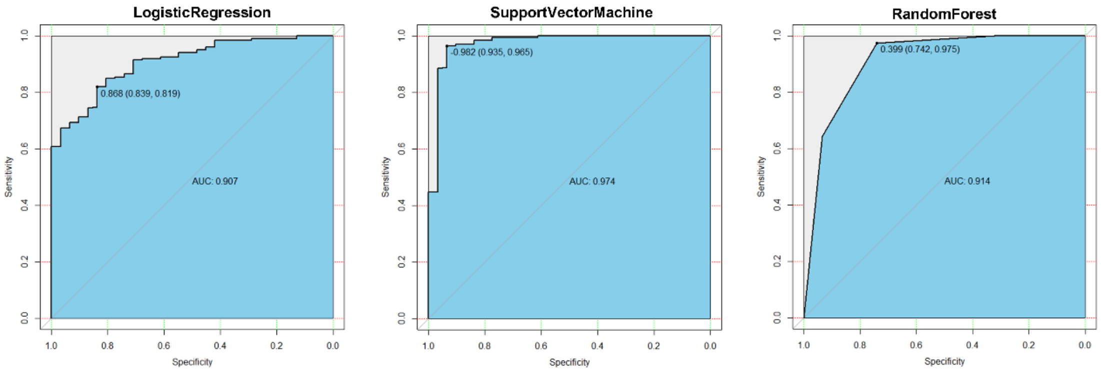

Supplement: Supplementary file 1 [file Data_Sheet_1.pdf]
